# Social Determinants Associated With Exposure to Childhood Parental Bereavement and Subsequent Risk for Psychiatric Disorders

**DOI:** 10.1001/jamanetworkopen.2022.39616

**Published:** 2022-10-31

**Authors:** Christy A. Denckla, Natalie M. Averkamp, Natalie Slopen, Ana Lucia Espinosa Dice, David Williams, M. Katherine Shear, Karestan C. Koenen

**Affiliations:** 1 Department of Social and Behavioral Sciences, Harvard TH Chan School of Public Health, Boston, Massachusetts; 2 Department of Biostatistics, Harvard TH Chan School of Public Health, Boston, Massachusetts; 3 Department of Epidemiology, Harvard TH Chan School of Public Health, Boston, Massachusetts; 4 Department of Sociology, Harvard University Faculty of Arts and Sciences, Cambridge, Massachusetts; 5 Columbia School of Social Work, New York, New York

## Abstract

**Importance:**

Prevalence of childhood parental death varies by race and ethnicity and socioeconomic status, yet whether similar variation persists in the association with lifetime psychiatric disorder is unknown.

**Objective:**

To assess whether race and ethnicity and parental educational attainment are associated with the risk of death of a parent; to determine whether the risk for lifetime psychiatric disorder associated with death of a parent was moderated by race and ethnicity and highest parental educational attainment; and to examine a potential intersection of race and ethnicity with parental educational attainment in the risk of lifetime psychiatric disorder associated with death of a parent.

**Design, Setting, and Participants:**

This retrospective cohort study used data from the National Comorbidity Study: Adolescent Supplement (NCS-A), 2001 to 2004. Participants included youth aged 13 to 18 years, restricted to Black, Hispanic, and White youth due to power limitations. Data were analyzed from February 26, 2021, to April 21, 2022.

**Exposure:**

Death of a parent during childhood.

**Main Outcomes and Measures:**

The primary study outcome was any lifetime *Diagnostic and Statistical Manual of Mental Disorders (Fourth Edition)* psychiatric disorder, assess via assessed via structured clinical interviews.

**Results:**

Among 9501 youth (mean [SD] age, 15.2 [1.5] years; 50.9% female), including 511 youth who had experienced parental death and 8990 youth who had not, the cumulative hazard of parental death by age 18 years was approximately doubled for Hispanic (10.1%; 95% CI, 6.9%-14.7%) and Black (14.0%; 95% CI, 10.6%-18.4%) youth compared with White youth (6.0%; 95% CI, 4.7%-7.8%). Similar patterns were noted by parental educational attainment: the cumulative hazard of parental death for youth of parents with less educational attainment was nearly double (10.1%; 95% CI, 8.1%-12.6%) compared with youth of parents with more education (6.6%; 95% CI, 5.2%-8.4%). Death of a parent was positively and significantly associated with risk of any lifetime psychiatric disorder (aOR, 1.34; 95% CI, 1.03-1.75) compared with youth who had not experienced death of a parent. However, this association was not moderated by race and ethnicity (aOR, 1.05; 95% CI, 0.58-1.92) or parental educational attainment (aOR, 1.19; 95%, 0.70-2.04), although power analyses suggest that larger sample sizes are needed.

**Conclusions and Relevance:**

In this cross-sectional study, Black and Hispanic youth experienced elevated parental death compared with White youth, yet the risk for any lifetime psychiatric disorder after parental death was not significantly moderated by race and ethnicity or parental education. Both individual- and population-level interventions may be needed to address the increased risk of psychiatric disorders, although additional studies with larger sample sizes are needed.

## Key Points

**Question:**

Are race and ethnicity and parental education associated with risk of parental death and psychiatric disorder?

**Findings:**

In this cohort study using National Comorbidity Study: Adolescent Supplement (NCS-A) data, the prevalence of parental death among Black and Hispanic youth was approximately twice that of White youth. Considered separately, neither race and ethnicity nor parental education moderated the significant association between bereavement and lifetime risk of psychiatric disorder; however, when considered jointly, variation in the association was noted.

**Meaning:**

These findings suggest that Black and Hispanic youth have a higher burden of parental death compared with White youth, yet lifetime risk of psychiatric disorder was similarly elevated.

## Teaser

This cohort study assesses associations of race and ethnicity, as well as parental education, with risk of parental death and subsequent psychiatric disorders in US youth.

## Introduction

The COVID-19 pandemic has elevated rates of parental death in the US by up to 20%^1^ above the baseline bereavement prevalence of approximately 4% for children younger than age 18 years.^2^ Youth who have experienced parental death are at risk for prolonged grief disorder,^3,4^ depression, anxiety, and posttraumatic stress disorder.^5^ Accumulating evidence illustrates social constructs of race and ethnicity^6,7^ shape access to resources, impact social experiences, and may be associated with risk of the death of a parent. For example, prior work has found that Black children are 3 times more likely than White children to have a mother die by age 10 years,^8^ a pattern noted as well during the COVID-19 pandemic.^9,10^ However, how the same social constructs that shape exposure additionally influence downstream mental health outcomes among youth who have lost a parent remains an open question. Prior work found that exposure to adversities in childhood increases risk for psychiatric disorders across all classes of *Diagnostic and Statistical Manual of Mental Disorders* (Fourth Edition) (*DSM-IV*) disorders,^11,12^ but social disparities in rates of onset of disorders in response to adversity are less consistent,^13^ such that risk after exposure is not uniformly elevated across all race and ethnicities.

Emerging consensus in disparities research recognizes that social determinants of health intersect by operating both as main effects and together conditioned across domains.^14^ In addition to race and ethnicity, parental educational attainment is also associated with health outcomes, including mortality.^15^ To better characterize the intersecting associations of social determinants of health, including race and ethnicity and parental educational attainment, with parental death and psychiatric risk among youth, we apply an intersectional health disparities framework to analyses of childhood parental health and lifetime psychiatric disorders in the National Comorbidity Survey: Adolescent Supplement (NCS-A) study, a representative cohort weighted for representation on a range of sociodemographic variables.^16,17^ In so doing, we aim to answer 3 questions relevant to population health, as well as disparities research more broadly: (1) are race and ethnicity and parental educational attainment associated with exposure to childhood parental death among youth aged 13 to 18 years? (2) do these same determinants moderate risk for psychiatric disorder among youth? and (3) do race and ethnicity and parental education, when considered jointly, explain variation in psychiatric disorders among youth?

## Methods

For this cohort study, recruitment and consent procedures were approved by the Human Subjects Committees of the University of Michigan and Harvard Medical School. Investigators completed an Agreement for the Use of Confidential Data with the Interuniversity Consortium for Political and Social Research at the University of Michigan. Written informed consent was obtained from youth’s legal guardians, and written assent was obtained from youth. Further details on sample can be found in the eMethods in the Supplement. This study is reported following the Strengthening the Reporting of Observational Studies in Epidemiology (STROBE) reporting guideline.

### Sample

Prior publications have reported detailed NCS-A study procedures.^18^ In brief, the NCS-A is a national survey of psychiatric disorders added on to the National Comorbidity Survey replication^19^ and was designed to estimate the prevalence of *DSM-IV*^12^ disorders among children and adolescents aged 13 to 18 years living in the United States. Data collection was conducted between February 2001 and January 2004 among English-speaking adolescents aged 13 to 18 years.

### Measures

#### Parental Death and Bereavement

Parental death and youth age at parental death were queried in the Adolescent Supplement interview via these items: “is your biological father still living?” and “is your biological mother still living?” Response options included yes, no, refused to answer, and do not know. When parental death was endorsed, follow up questions included “how old were you when he/she died?” Adolescents with responses of do not know or refuse to say (94 adolescents) were designated not bereaved, given that the primary research question related to the experience of parental bereavement among youth, which is logically conditioned on knowing that a parent had died.

#### Psychiatric Diagnostic Assessment

A modified version of the World Health Organization Composite International Diagnostic Interview,^20^ a structured clinical assessment instrument, was administered by trained lay interviewers. This study focuses on a single outcome of any lifetime *DSM-IV* psychiatric disorder, which includes the full range of possible psychiatric disorders referenced in the *DSM-IV* (eMethods in the Supplement). Onset information for some disorders, including distress disorders, was only available from parent report. Among youth with a deceased biological parent, 295 of 536 (55.0%) of the parents provided data, compared with 64.0% of children who had not experienced parental death.

#### Social Determinants

Race and ethnicity were self-defined by participants based on the following question, “Which of the following best describes your race: American Indian, Alaska Native, Asian, black or African American, Native Hawaiian, Pacific Islander, or white?” Race and ethnicity were then recoded as a single variable with categories of Black, Hispanic, White, and other. Parental educational attainment was defined as the highest education level either parent completed, including less than high school, high school diploma, some college, or college graduate. To maintain adequately powered cell sizes for between-group comparisons, parental education was condensed from 4 categories to 2 (low and high) with less than high school and high school diploma combined in the low or less educated group, and some college and college graduate in the high or more educated group.

### Statistical Analysis

There was no missingness in study variables, including race and ethnicity, parental education, parental income, age, and sex. Descriptive analyses compared bereaved and nonbereaved groups across race and ethnicity (Black, Hispanic, and White), parental education (high and low), and demographic covariates. Then we estimated the cumulative risk for parental death separately by parental education levels (low vs high) and race and ethnicity (Black, Hispanic, and White) using a Kaplan-Meier life table. An abridged life table was constructed in 3-year segments (ages 0-5, 6-13, and 14-18 years), reflecting risk of bereavement before reaching age *t*. The survival function, *S*(*t*), was estimated using the Kaplan-Meier estimator, and the cumulative hazard is given by −log *S*(*t*). A log-rank test was then used to assess whether there was a statistically significant difference among the survival curves. The R package *survey*^21^ (R Project for Statistical Computing) was used to implement survey weights that account for the complex survey design.

We explored statistical power analysis^22^ for interaction effects in cross-sectional data sets (eMethods in the Supplement). We found that we had greater than 80% power to identify a correlation between *X*1 × *X*2 and *Y* of at least 0.1, but were likely underpowered to identify correlations between *X*1 × *X*2 and Y of less than 0.1, which may be expected given the low observed correlations between our binary study variables.

Next, we estimated the association of bereavement with risk for any lifetime psychiatric disorder, with youth age at parental death, youth sex, youth race and ethnicity, parental education, parental income, and a race and ethnicity × education interaction term covaried in our primary model, given the significant between-group differences among these demographic variables (eTable 1 in the Supplement). We separately tested for modification by race and ethnicity and parental education in the association between bereavement and lifetime psychiatric disorder risk by entering interaction terms between bereavement and these variables into main effects models. Given power limitations, we adjusted the primary model slightly, dichotomizing the 3-category race and ethnicity variable into White vs Black and Hispanic and excluding the race and ethnicity × education interaction term covariate due to its having been found not to be significant. Models are interpreted with White youth without a parent who died as the reference group, given this group had the largest cell sizes. Regression results are reported using odds ratios (ORs).

In the final stage, predicted probabilities of lifetime psychiatric disorder were calculated for all possible combinations of race and ethnicity and high vs low educational attainment, while setting bereavement to positive, age to the overall sample mean, sex to female, and poverty category to lowest. Then, to make relative comparisons across race and ethnicity groups, we proceeded with standard percentage change calculations.

Statistical significate was set at 2-sided *P* < .05. Analyses were conducted in R statistical software version 4.1.0 (R Project for Statistical Computing. Data were analyzed from February 26, 2021, to April 21, 2022.

## Results

Among the 9501 youth (mean [SD] age, 15.2 [1.5] years; 50.9% female) in the analytic sample (eFigure 1 in the Supplement), 8990 youth had not experienced parental death and 511 youth had experienced parental death. While the overall prevalence of bereavement among Black, Hispanic, and White youth was 5.4%, the proportion of youth who had experienced parental death varied by race and ethnicity and parental educational attainment. Table 1 displays stratified proportions of the total cohort sample across race and ethnicity, parental education levels, and bereavement status, illustrating that parental death was unevenly distributed across race and ethnicity and education level categories.

**Table 1.** Demographic Comparisons Between Youth With and Without Parental Bereavement Stratified by Race and Ethnicity

Parental education^a^	Youth, No. (%) (N = 9501)							
	Bereaved (n = 511)				Nonbereaved (n = 8990)			
	Black	Hispanic	White	Total	Black	Hispanic	White	Total
High	55 (10.7)	32 (6.3)	132 (25.8)	219 (42.8)	700 (7.8)	632 (7.0)	3491 (38.8)	4823 (53.6)
Low	126 (24.7)	76 (14.9)	90 (17.6)	292 (57.1)	1072 (11.9)	1174 (13.1)	1921 (21.4)	4167 (46.4)
Total	181 (35.4)	108 (21.1)	222 (43.4)	511 (100)	1772 (19.7)	1806 (20.1)	5412 (60.2)	8990 (100)

^a^ Low education includes high school diploma or less; high education, some college or more.

**Figure 1.**
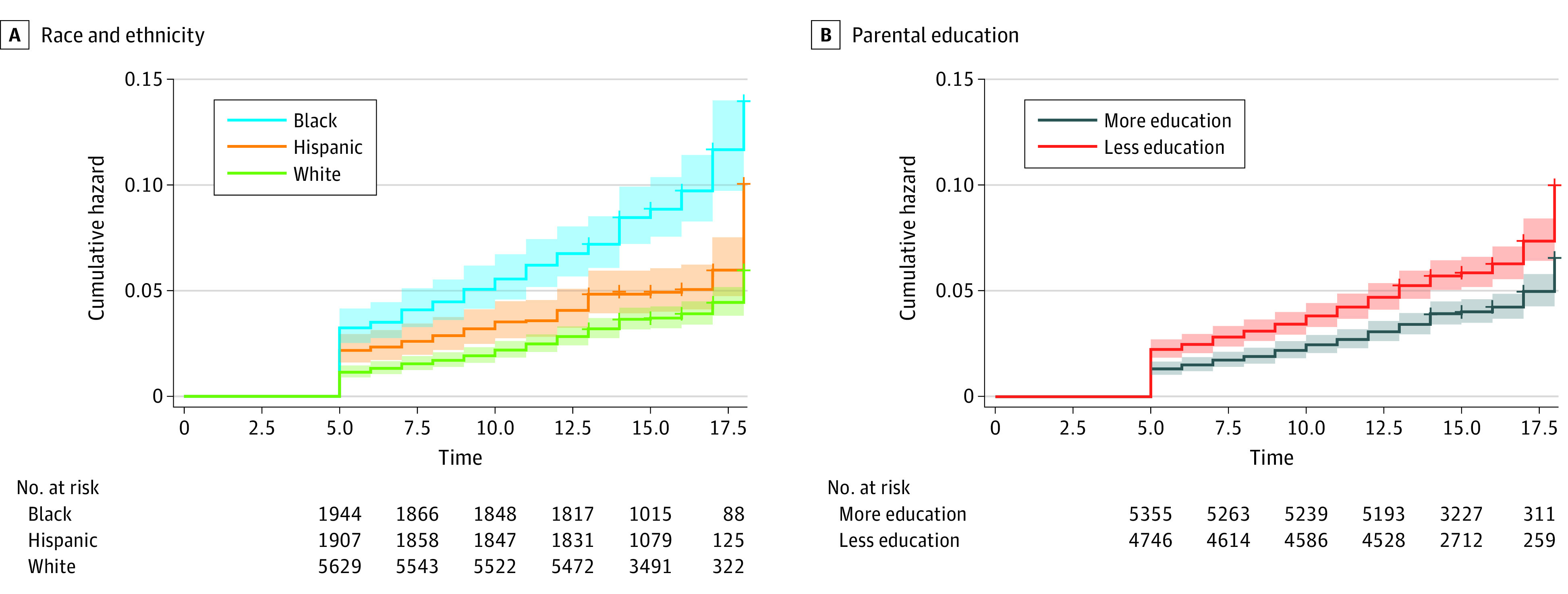
Cumulative Hazard Plots for Any Parental Death Shading indicates 95% CI.

Table 2 reports cumulative hazard of parental death segmented into age categories by race and ethnicity and parental education. The abridged life table demonstrates that as early as age 5 years, the cumulative hazard for parental death was almost 3 times greater for Black youth compared with White youth. When youth reached age 18 years, the cumulative hazard for parental death for White adolescents was 6.0% (95% CI, 4.7%-7.8%), whereas the cumulative hazard for Black youth (14.0%; 95% CI, 10.6%-18.4%) and Hispanic youth (10.1%; 95% CI, 6.9%-14.7%) was approximately double. Differences among the 3 race categories in the survival curves at any 1 time for parental death were significant (log-rank test χ^2^ = 78.6, *P* < .001). Disparities were also found in the cumulative hazard for risk of parental death by parental education levels. As early as age 5 years, children with parents who had lower educational attainment were twice as likely to experience parental death compared with their counterparts with parents of higher educational attainment. When youth reached age 18 years, the cumulative hazard for parental death for youth with more educated parents was 6.6% (95% CI, 5.2%-8.4%), whereas the cumulative hazard for youth with less educated parents was 10.1% (95% CI, 8.1%-12.6%). Figure 1 presents cumulative hazard plots segmented by race and ethnicity and parental education levels. Finally, we found that cumulative hazard for parental death by race and ethnicity varied by sex of the deceased parent. eTable 2 in the Supplement displays cumulative hazard of risk for parental death separately by paternal and maternal death, demonstrating higher cumulative hazard for paternal death specifically among Black youth compared with Hispanic or White youth (eFigure 2 in the Supplement).

**Table 2.** Cumulative Risk of Parental Bereavement Before Youth Reach Age *t*

Age, y	Cumulative hazard (95% CI), %^a^				
	Parental education^b^		Race and ethnicity^c^		
	Low	High	Black	Hispanic	White
5	0.02 (0.02-0.03)	0.01 (0.01-0.02)	0.03 (0.03-0.04)	0.02 (0.02-0.03)	0.01 (0.01-0.02)
13	0.05 (0.05-0.06)	0.03 (0.03-0.04)	0.07 (0.06-0.09)	0.05 (0.04-0.06)	0.03 (0.03-0.04)
18	0.10 (0.10-0.13)	0.07 (0.05-0.08)	0.14 (0.12-0.18)	0.10 (0.07-0.15)	0.06 (0.05-0.08)

^a^ Cumulative hazard with 95% CI was calculated using the nonparametric life table approach, given by −log *S*(*t*). The Kaplan-Meier estimator was used to estimate the survival function.

^b^ Low education includes high school diploma or less; high education, some college or more.

^c^ Differences among the survival curves among the 3 race categories at least at 1 time for any parental death were significant (log-rank test χ^2^ = 78.6; *P* < .001).

Next, we examined the association between exposure to parental death and youth lifetime psychiatric disorder. First, we found an association of any parental death with risk for any lifetime psychiatric disorder after adjusting for youth sex, youth age at survey, youth race and ethnicity, parental income, parental education, and race and ethnicity × education interaction (adjusted odds ratio [aOR], 1.34; 95% CI, 1.03-1.75) (Table 3). Black and Hispanic youth who had experienced parental death evidenced significantly elevated risk for any psychiatric disorder compared with White youth who had not experienced parental death (Black youth: aOR, 1.42; 95% CI, 0.91-2.21; Hispanic youth: aOR, 1.91; 95% CI, 1.30-2.80). However, the interaction term bereavement × race and ethnicity was not significant (aOR, 1.06; 95% CI, 0.58-1.92).

**Table 3.** Risk of Any Psychiatric Disorder by Bereavement, Race and Ethnicity, and Parental Education^a^

Variable	Youth without bereavement			Youth with bereavement		
	With disorder, No.	Without disorder, No.	OR (95% CI)	With disorder, No.	Without disorder, No.	OR (95% CI)
Race and ethnicity						
Black	930	842	1.05 (0.75-1.49)	66	42	1.42 (0.91-2.21)
Hispanic	1004	802	1.42 (1.05-1.92)^b^	112	69	1.91 (1.30-2.80)^b^
White	2724	2724	1 [Reference]	132	90	1.34 (1.03-1.75)^b^
Parental education^c^						
High	2576	2555	1 [Reference]	132	95	1.34 (1.03-1.75)^b^
Low	2407	2049	1.27 (1.02-1.59)^b^	191	118	1.71 (1.29-2.26)^b^

Abbreviation: OR, odds ratio.

^a^ Models above include youth sex, youth age, family income, education, race and ethnicity, and education × race and ethnicity as covariates.

^b^ We excluded an interaction term between bereavement and race and ethnicity due to its not being significant in preliminary analyses (OR, 1.05; 95% CI, 0.58-1.92). We excluded an interaction term between bereavement and education due to its not being significant either (OR, 1.19; 95% CI, 0.70-2.04).

^c^ Low education includes high school diploma or less; high education, some college or more.

Similar patterns were noted in separate models examining the differential association of bereavement with risk for any lifetime psychiatric disorder by parental education (Table 3). We observed elevated risk among youth who had not experienced parental death with parents with lower education (aOR, 1.27; 95% CI, 1.02-1.59), as well as among youth had experienced parental death regardless of parental education level (higher: aOR, 1.34; 95% CI, 1.03-1.75; lower: aOR, 1.71; 95% CI, 1.29-2.26), compared with White youth who had not experienced parental death. The interaction term between parental death and parental education was not significant (aOR, 1.19; 95% CI, 0.70-2.04).

**Figure 2.**
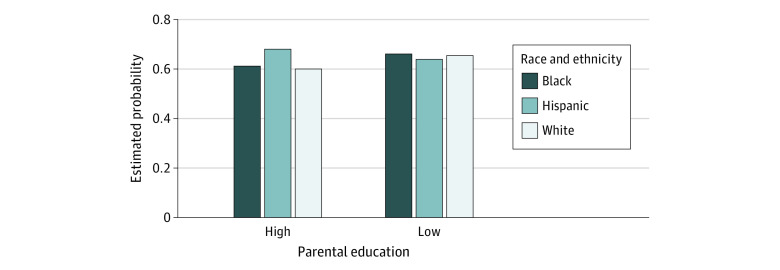
Estimated Probability of Any Psychiatric Disorder Among Youth With Parental Bereavement by Parental Education and Race and Ethnicity Parental education was dichotomized as low, completed high school or less; high, some college or more.

To test our hypotheses regarding the potential combined associations of race and ethnicity and parental education with risk for any psychiatric disorder among youth who had experienced parental death, we calculated predicted probabilities with summary models set with parental death to positive, age to the overall sample mean, sex to female, and poverty category to lowest (Figure 2; eTable 3 in the Supplement). Although differences between groups were relatively small, the predicted probability of any psychiatric disorder among Hispanic youth who had experienced parental death was highest among those with parents with higher levels of education 0.68 (95% CI, 0.60-0.75) compared with youth with parents with lower education (0.64; 95% CI, 0.56-0.72). Opposite patterns were noted for Black and White youth, for whom higher parental education was associated with lower probability of psychiatric disorder (Black: 0.61; 95% CI, 0.50-0.71; White: 0.60; 95% CI, 0.530, 0.67) compared with youth with lower parental education (Black: 0.66; 95% CI, 0.59-0.73; White: 0.67; 95% CI, 0.59-0.72). Percentage change in estimated probabilities showed that Black and White youth who had not experienced parental death with higher levels of parental education were less likely to experience any psychiatric disorder (Black: 8.2%; 95% CI, −12.6% to 28.9%; White: 9.3%; 95% CI: −6.6% to 25.3%) compared with race-matched youth who had experienced parental death and whose parents had lower education levels. However, this protective association was not evident for Hispanic youth, who were 5.9% more likely to experience any psychiatric disorder compared with Hispanic youth with parents with less education.

## Discussion

This cohort study using data from the NCS-A found that social determinants of health, including race and ethnicity and parental education, were associated with risk of parental death. These results are consistent with prior work^23^ showing the cumulative hazard for parental death for Black and Hispanic youth was nearly double that for White youth. Our findings augment the literature by demonstrating that parental education was also significantly associated with parental death. Youth reporting fewer years of parental educational attainment were nearly twice as likely to report the death of a biological parent compared with youth with parents in the higher educated group. Bereavement exposure disparities intersect across educational attainment and race and ethnicity status, impacting the developmental social ecosystem in which children grow and stand to thrive.^24^ This leaves important questions to be addressed in prospective studies, including understanding the root causes of higher bereavement in Black and Hispanic youth.

This study extends the literature by investigating how theses social determinants are associated with psychiatric disorders among youth with parental death, considering race and ethnicity and parental education level. Consistent with prior work,^8,23,25^ we found evidence for an association of parental death with risk for any lifetime psychiatric disorder among youth. However, we did not find evidence for a significant interaction of bereavement with race and ethnicity or bereavement with parental education in risk for any lifetime psychiatric disorder. Notably, our power analyses suggested that larger sample sizes would be needed to detect associations between our interacting variables and psychiatric outcome at lower effect sizes, warranting caution in interpreting a lack of significant interaction. Of note, these results do not include symptoms of prolonged grief disorder, so outcomes may be underestimated. Given that Black and Hispanic youth have elevated rates of parental death, the greater burden of mental disorders among Black and Hispanic children may be partially associated with bereavement, consistent with the Surgeon General’s 2021 Advisory report.^26^

When we considered the intersection of race and ethnicity with parental education in shaping risk for lifetime psychiatric disorder among youth with parental death, we found that the combination of parental education with race and ethnicity explained variation in risk for any lifetime psychiatric disorder in opposite directions for Hispanic youth compared with Black and White youth. The protective association among Black and White youth of higher parental education was not noted for Hispanic youth. Immigration history might be one possible explanation of this difference, based on a 2008 study^27^ showing that changes in health status among immigrants covaried with education and length of residence, in some cases worsening with length of stay. Future research on the intersections of social determinants of health is necessary to reduce the population-level burden of postbereavement health conditions.

### Limitations

Several limitations should be considered when interpreting study findings. First, missing data on timing of disorder onset for a significant proportion of youth with parental limited capacity to infer temporal associations between parental death and psychiatric disorder risk, nor were we able to control for parental psychiatric disorder as a confounder. Also, these cross-sectional data did not capture chronicity or persistence of mental disorders, an important consideration in health disparity research.^28^ Second, we were unable to consider the association of compounded adversity secondary to parental death nor its association on mental health owing to limitations in linking parental death to disorder onset. Third, while the size of the study sample of adolescents with parental death exceeded that typically seen in prior research, we were still underpowered to consider a wider range of socioeconomic variables, including making comparisons among Alaska Native, American Indian, or Asian groups. Further research is needed in adequately powered data sets with representation from Alaska Native, American Indian, and Asian youth. Relatedly, we did not have data on mixed race and ethnicity families, limiting consideration of the growing population of mixed race and mixed ethnicity youth. Fourth, constraining bereavement to the death of a biological parent could misestimate the association of bereavement of extended family or nonbiological caregivers with mental health outcomes, especially in settings of diverse family structure.^29^ Fifth, we classified the 94 youth who stated that they did not know if their biological parent had died as not bereaved, thus potentially misclassifying youth with parental death as nonbereaved. However, given our research question and youth bereavement status logically conditioned on awareness of parental death, we opted to classify these individuals as nonbereaved rather than impute or remove from analyses. Finally, given that nearly half of the youth who were bereaved did not have a completed parental self-administered questionnaire, it is possible that symptoms could have been underreported. While similar proportions of youth who were bereaved (55.0%) and those who were not bereaved (64.0%) had parent-completed self-administered questionnaire data, suggesting comparability between these groups, caution is warranted, given the potential for differential nonreporting.

## Conclusions

In this cohort study of youth reporting the death of a biological parent, parental bereavement was associated with elevated risk for any lifetime psychiatric disorder. Black and Hispanic youth experienced elevated rates of parental death compared with White youth, as did youth with parents with lower educational attainment. Results underscore the need for population level, prospective representative data sets to more precisely characterize the association between social determinants, parental bereavement, and mental health. Individual-level and population-level interventions may be needed to improve health equity for all youth, and especially among Black and Hispanic youth who carry excess population-level burden of parental death compared with White youth.

## Notes

Supplement.
eMethods.
**eTable 1.** Descriptive Demographics and Between-Group Comparisons Among Youth With and Without Parental Death in the NSC-A Cohort **eTable 2.** Cumulative Risk of Parental Bereavement Before the Adolescent Reaches Age *t*, by Race and Ethnicity, Reported Separately for Maternal, Paternal, and any Parental Loss **eTable 3.** Estimated Probabilities of any *DSM-IV* Psychiatric Disorder Stratified by Race and Ethnicity and Parental Education Among Youth With Parental Death **eFigure 1.** Analytic Sample Participant Flowchart **eFigure 2.** Cumulative Hazard Plots Stratified by Race and Ethnicity, Reported Separately by Type of Loss
eReferences.
