# Supplementary material for: Social Determinants Associated With Exposure to Childhood Parental Bereavement and Subsequent Risk for Psychiatric Disorders
Source: JAMA Netw Open. 2022 Oct 31;5(10):e2239616. doi: 10.1001/jamanetworkopen.2022.39616 (PMC9623444; doi:10.1001/jamanetworkopen.2022.39616)
Supplement: Supplement. — eMethods. eTable 1. Descriptive Demographics and Between-Group Comparisons Among Youth With and Without Parental Death in the NSC-A Cohort eTable 2. Cumulative Risk of Parental Bereavement Before the Adolescent Reaches Age t, by Race and Ethnicity, Reported Separately for Maternal, Paternal, and any Parental Loss eTable 3. Estimated Probabilities of any DSM-IV Psychiatric Disorder Stratified by Race and Ethnicity and Parental Education Among Youth With Parental Death eFigure 1. Analytic Sample Participant Flowchart eFigure 2. Cumulative Hazard Plots Stratified by Race and Ethnicity, Reported Separately by Type of Loss eReferences. [file jamanetwopen-e2239616-s001.pdf]

Supplemental Online Content

Denckla CA, Averkamp NM, Slopen N, et al. Social determinants associated with exposure to childhood parental bereavement and subsequent risk for psychiatric disorders. *JAMA Netw Open*. 2022;5(10):e2239616.
doi:10.1001/jamanetworkopen.2022.39616

eMethods.
eTable 1. Descriptive Demographics and Between-Group Comparisons Among Youth With and Without Parental Death in the NSC-A Cohort
eTable 2. Cumulative Risk of Parental Bereavement Before the Adolescent Reaches Age *t*, by Race and Ethnicity, Reported Separately for Maternal, Paternal, and any Parental Loss
eTable 3. Estimated Probabilities of any *DSM-IV* Psychiatric Disorder Stratified by Race and Ethnicity and Parental Education Among Youth With Parental Death
eFigure 1. Analytic Sample Participant Flowchart
eFigure 2. Cumulative Hazard Plots Stratified by Race and Ethnicity, Reported Separately by Type of Loss
eReferences.

This supplemental material has been provided by the authors to give readers additional information about their work.

© 2022 Denckla CA et al. *JAMA Network Open.*

eMethods.

Sample

NCS-A was based on a dual frame design in which one sample was recruited from the NCS-R households, and a second recruited from a representative sample of schools in the same communities as the NCS-R household [1] . The total sample, combining households and schools, included 10,123 youth. The household sample consisted of 879 school- attending adolescents recruited from the NSC-R household sample (response rate of 85.9%). The school sample included 9244 adolescents recruited from 320 representative schools geographically proximal to NCS-R households. The response rate for adolescents recruited from schools was 74.7%. Interviewers obtained written consent from a parent or guardian, and written informed assent from the adolescent. Participants were remunerated $50 as a token of appreciation. The Human Subjects Committees of both Harvard Medical School (HMS) and the University of Michigan approved recruitment, consent, and field procedures. One parent or guardian completed paper-and-pencil self-administered questionnaires about the study adolescent's mental health and developmental history while their children were completing their interview separately. Response rates for the adult questionnaires were 82.5% in the household sample, and 83.7% in the school sample. Weighting procedures are described previously [1]. Briefly, household sample cases were doubly-weighted by combining weights derived for the NCS-R household sample taking into account residual discrepancies between sample and population sociodemographic and geographic discrepancies with those derived after adjustment for differential probability of selection of adolescents as a function of other adolescents in the household. Present analyses were limited to three race/ethnicity groups: White, Black, and Hispanic. Participants who

© 2022 Denckla CA et al. *JAMA Network Open.*

identified as ‘other’ race (n=622 total) were not included in the present analytic sample due to lack of power for planned analyses.

Measures

Psychiatric Diagnostic Assessment

Both adolescents and parents provided diagnostic information. Parents completed a self-administered questionnaire to collect additional information about adolescent symptoms of Major Depressive Disorder/Dysthymia, Attention Deficit/Hyperactivity Disorder/Oppositional Defiant Disorder, and Conduct Disorder [2]. 55.0% of bereaved youth had data from the parent completed self-administered questionnaire, and 64.0% of non-bereaved youth had parent completed self-administered questionnaire data, suggesting comparability between these two groups. Parent and adolescent reports were combined at the symptom level using an “or” rule, such that a symptom was considered present if it was endorsed by either respondent based on prior studies demonstrating that such an approach improves diagnostic accuracy [3] . Diagnostic hierarchy and organic exclusion rules were used, similar to procedures in prior investigations in the NCS-A dataset [4].

Power Analysis

We explored statistical power for this analysis using the InteractionPowerR package in R [5]. Assuming a sample size of N=10,000 with exposure prevalence of 5.0%, interacting variable prevalence of 40.0%, and outcome prevalence of 50.0%, we draw on 1000 iterations and alpha=0.05 to explore statistical power. Based on exploratory analyses, we explore scenarios with small correlations between bereavement (X1), race/education (X2), and lifetime psychiatric disorder (Y). We identify a few plausible scenarios in which we are sufficiently powered to identify the interaction term of interest. For example, assuming a correlation between X1 and Y of 0.1, a

© 2022 Denckla CA et al. *JAMA Network Open.*

correlation between X2 and Y of 0.1, and a correlation between X1 and X2 of 0.08, we have greater than 80% power to identify a correlation between X1*X2 and Y of at least 0.1. Alternatively, assuming a correlation between X1 and Y of 0.05, a correlation between X2 and Y of 0.05, and a correlation between X1 and X2 of 0.05, we have greater than 80% power to identify a correlation between X1*X2 and Y of at least 0.1. We are likely underpowered to identify correlations between X1*X2 and Y of less than 0.1, which may be expected given the low observed correlations between our binary study variables.

© 2022 Denckla CA et al. *JAMA Network Open.*

eTable 1. Descriptive Demographics and Between-Group Comparisons Among Youth With and Without Parental Death in the NSC-A Cohort

	Bereaved (n = 536) (n, %)	Not bereaved (n = 9,587), (n, %)	Total (n = 10,123), (n, %)	*p*-value[b]
Sex				
Male	245 (45.7)	4708 (49.1)	4953 (48.9)	0.126
Female	291 (54.3)	4879 (50.9)	5170 (50.9)	
Age at survey	15.35 (13-18)	15.17 (13-18)	15.178 (13-18)	0.005
Race				
Black	181 (33.8)	1772 (18.5)	1953 (19.3)	
Hispanic	108 (20.1)	1806 (18.8)	1914 (18.9)	< 0.001
White	222 (41.4)	5412 (56.5)	5634 (55.7)	
Other	25 (4.7)	597 (6.2)	622 (6.1)	
Household poverty (ratio of income to poverty line)[a]				
≤ 1.5 (low)	119 (22.2)	1598 (16.7)	1717 (17.0)	0.008
≤ 3 (low-average)	101 (18.8)	1922 (20.0)	2023 (20.0)	
≤ 6 (high-average)	162 (30.2)	2939 (30.7)	3101 (30.6)	
> 6 (high)	154 (28.7)	3128 (32.6)	3282 (32.4)	
Parent Education				
< HS	123 (22.9)	1561 (16.3)	1684 (16.6)	< 0.001
HS	186 (37.7)	2895 (30.2)	3081 (30.4)	
Some College	95 (17.7)	1903 (19.8)	1998 (19.7)	
College grad	132 (24.6)	3228 (33.7)	3360 (33.2)	

Notes: [a] Household poverty is reported as the ratio of income to the poverty line where lower ratios represent lower income. [b]Significant p-value for age estimated using t-tests assuming equal variance; Pearson's chi-squared test used for all other variables.

© 2022 Denckla CA et al. *JAMA Network Open.*

eTable 2. Cumulative Risk of Parental Bereavement Before the Adolescent Reaches Age *t*, by Race and Ethnicity, Reported Separately for Maternal, Paternal, and any Parental Loss

	Maternal loss			Paternal loss			Any parental loss		
Age	Black	Hispanic	White[a]	Black	Hispanic	White	Black	Hispanic	White
5	0.007 (0.004, 0.012)	0.004 (0.002, 0.008)	0.003 (0.002, 0.004)	0.027 (0.020, 0.035)	0.018 (0.013, 0.025)	0.009 (0.007, 0.012)	0.032 (0.025, 0.042)	0.022 (0.016, 0.030)	0.011 (0.009, 0.015)
10	0.012 (0.008, 0.018)	0.011 (0.007, 0.017)	0.005 (0.004, 0.008)	0.045 (0.036, 0.055)	0.025 (0.019, 0.033)	0.017 (0.014, 0.020)	0.056 (0.046, 0.067)	0.035 (0.028, 0.045)	0.022 (0.018, 0.026)
15	0.024 (0.018, 0.032)	0.016 (0.011, 0.023)	0.009 (0.007, 0.012)	0.068 (0.057, 0.081)	0.033 (0.026, 0.043)	0.028 (0.024, 0.033)	0.089 (0.076, 0.104)	0.049 (0.040, 0.061)	0.037 (0.032, 0.043)
18	0.051 (0.028, 0.093)	0.046 (0.025, 0.083)	0.013 (0.008, 0.022)	0.091 (0.074, 0.112)	0.057 (0.037, 0.088)	0.048 (0.036, 0.064)	0.140 (0.106, 0.184)	0.101 (0.069, 0.147)	0.060 (0.047, 0.078)

Note: [a]Differences among the survival curves among the three race categories at least at one time for any parental loss (log-rank test X^2 = 78.6, p < 0.001), maternal loss (X^2 = 28.6, p < 0.001) and paternal loss (X2 = 62.1, p < 0.001)

© 2022 Denckla CA et al. *JAMA Network Open.*

eTable 3. Estimated Probabilities of any *DSM-IV* Psychiatric Disorder Stratified by Race and Ethnicity and Parental Education Among Youth With Parental Death

	Black	Hispanic	White
high education[a]	0.612 (0.503, 0.710)	0.680 (0.597, 0.753)	0.599 (0.529, 0.666)
low education	0.662 (0.586, 0.730)	0.640 (0.558, 0.715)	0.655 (0.587, 0.717)

Note: [a]Estimates are derived from a primary model in which bereavement is set to positive, age to the overall sample mean, sex to female, and poverty category to lowest.

© 2022 Denckla CA et al. *JAMA Network Open.*

eFigure 1. Analytic Sample Participant Flowchart

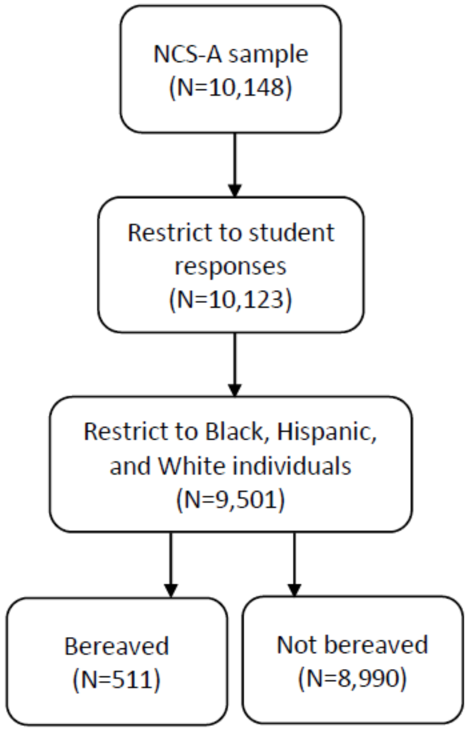

© 2022 Denckla CA et al. *JAMA Network Open.*

eFigure 2. Cumulative Hazard Plots Stratified by Race and Ethnicity, Reported Separately by Type of Loss[a]

A. Paternal Loss Only

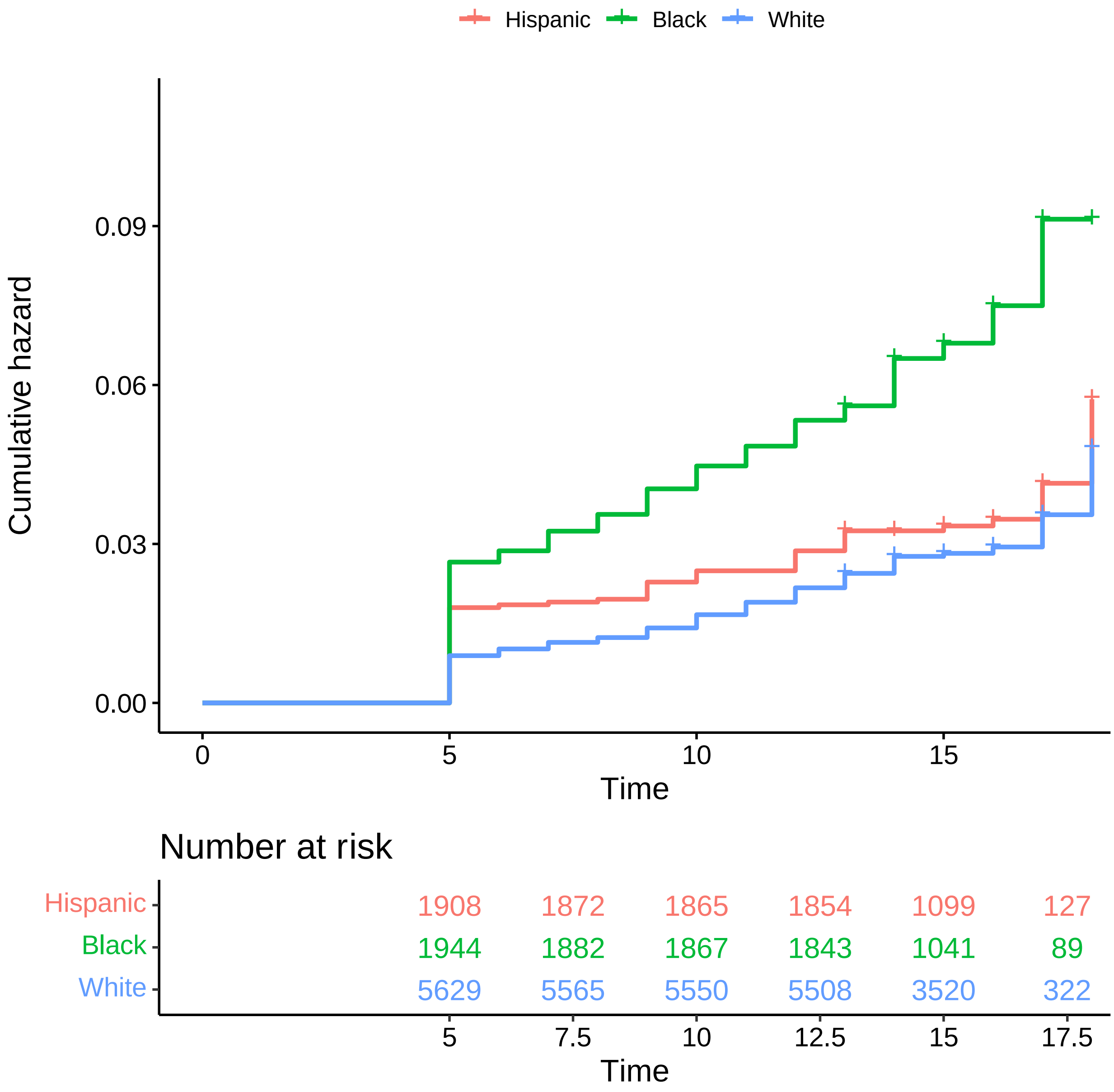

© 2022 Denckla CA et al. *JAMA Network Open.*

B. Maternal Loss Only

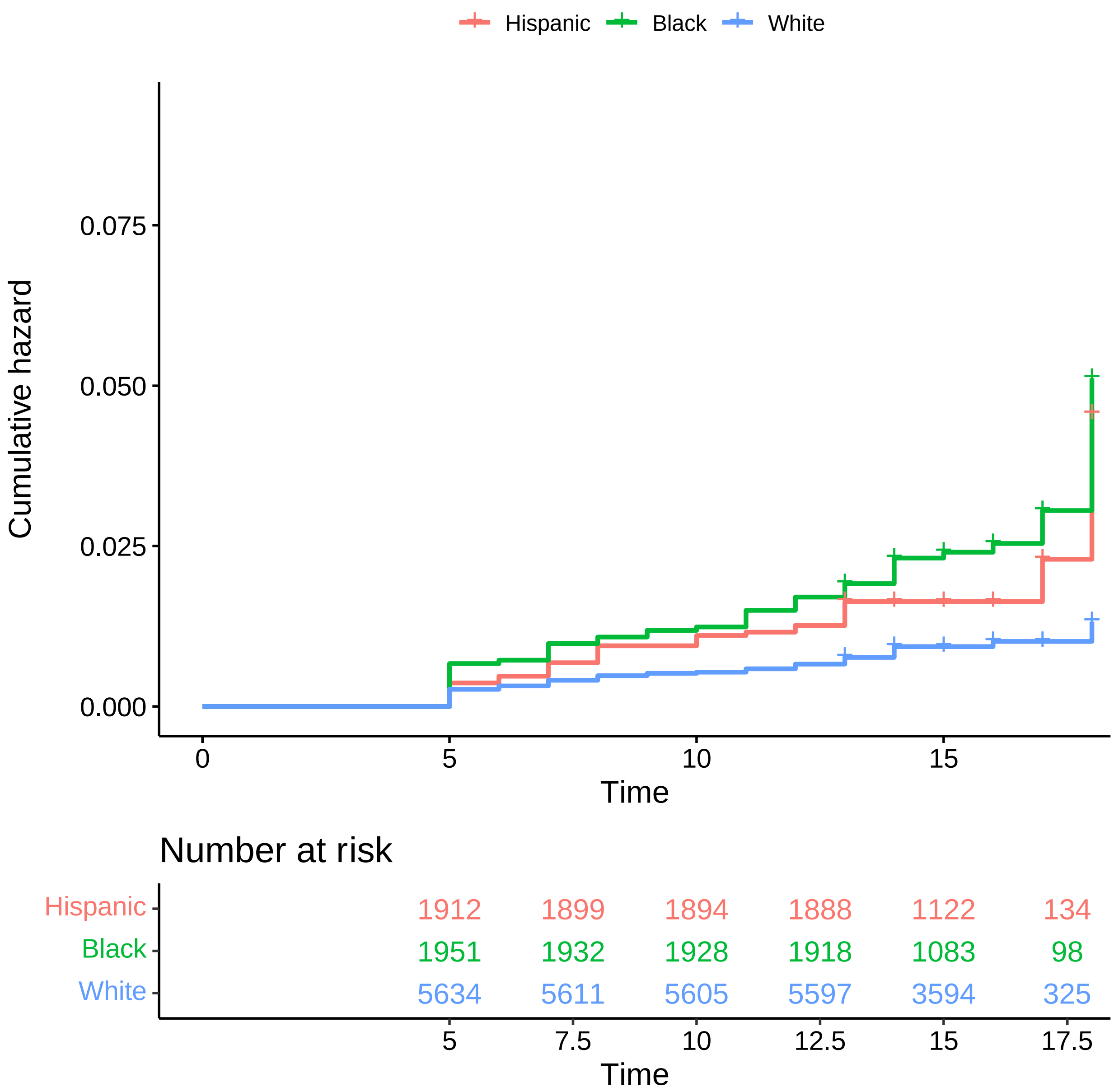

Note: [a]Hazards increase after age 15 because the data was recorded as a loss at 15 or older occurred, so for an 18 year old, any individual could have been bereaved anytime between the ages of 15 and 18. Hazards therefore reflect a 3-year time frame rather than the 1 year time frame depicted for children aged 5 to 15.

© 2022 Denckla CA et al. *JAMA Network Open.*

eReferences.

1. Kessler RC, Avenevoli S, Costello EJ, et al. National comorbidity survey replication adolescent supplement (NCS-A): II. Overview and design. *J Am Acad Child Adolesc Psychiatry*. 2009;48(4):380-385.

2. Bird HR, Gould MS, Staghezza B. Aggregating data from multiple informants in child psychiatry epidemiological research. *J Am Acad Child Adolesc Psychiatry*. 1992;31(1):78-85.

3. Kessler RC, Avenevoli S, Costello EJ, et al. Prevalence, persistence, and sociodemographic correlates of DSM-IV disorders in the National Comorbidity Survey Replication Adolescent Supplement. *Arch Gen Psychiatry*. 2012;69(4):372-380.

4. McLaughlin KA, Green JG, Gruber MJ, Sampson NA, Zaslavsky AM, Kessler RC. Childhood adversities and adult psychopathology in the National Comorbidity Survey Replication (NCS-R) III: associations with functional impairment related to DSM-IV disorders. *Psychol Med*. 2010;40(5):847-859.

5. Baranger DAA, Finsaas MC, Goldstein BL, Vize CE, Lynam DR, Olino TM. Tutorial: Power analyses for interaction effects in cross-sectional regressions. *psyarxiv.com*. Published online 2022. doi:10.31234/osf.io/5ptd7

© 2022 Denckla CA et al. *JAMA Network Open.*
